# Measuring consumer access, appraisal and application of services and information for dementia (CAAASI-Dem): a key component of dementia literacy

**DOI:** 10.1186/s12877-020-01891-3

**Published:** 2020-11-19

**Authors:** Kathleen Veronica Doherty, Hoang Nguyen, Claire E. A. Eccleston, Laura Tierney, Ron L. Mason, Aidan Bindoff, Andrew Robinson, James Vickers, Fran McInerney

**Affiliations:** Wicking Dementia Research and Education Centre, College of Health and Medicine, University of Tasmania, Hobart, Tasmania 7001 Australia

**Keywords:** Dementia, Literacy, Carer, Services, Measurement

## Abstract

**Background:**

The ability to locate, navigate and use dementia services and information, either for oneself or in providing care for others, is an essential component of dementia literacy. Despite dementia literacy being understood to be inadequate in many settings, no validated instrument exists to measure these elements. Here we describe the development and preliminary validation of the Consumer Access, Appraisal and Application of Services and Information for Dementia (CAAASI-Dem) tool.

**Methods:**

Items were adapted from existing health literacy tools and guided by discussion posts in the Understanding Dementia Massive Open Online Course (UDMOOC). Following expert review and respondent debriefing, a modified CAAASI-Dem was administered to UDMOOC participants online. On the basis of descriptive statistics, inter-item and item total correlations and qualitative feedback, this was further refined and administered online to a second cohort of UDMOOC participants. Exploratory factor analysis identified underlying factor structure. Items were retained if they had significant factor loadings on one factor only. Each factor required at least three items with significant factor loadings. Internal consistency of factors in the final model was evaluated using Cronbach’s alpha coefficients.

**Results:**

From a pool of 70 initial items with either a 5-point Likert scale (Not at all confident – Extremely confident; or Strongly agree – Strongly disagree) or a binary scale (Yes – No), 65 items were retained in CAAASI-Dem-V1. Statistical and qualitative analysis of 1412 responses led to a further 34 items being removed and 11 revised to improve clarity. The 31 item CAAASI-Dem-V2 tool was subsequently administered to 3146 participants, one item was removed due to redundancy and EFA resulted in the removal of an additional 4 items and determination of a five factor structure: *Evaluation and engagement; Readiness; Social supports; Specific dementia services*; and *Practical aspects*.

**Conclusions:**

The five factors and 26 constituent items in CAAASI-Dem align with functional, critical, and communicative aspects of dementia health literacy from the perspective of the carer. As a screening tool for people living with dementia and their carers, CAAASI-Dem potentially provides a means to determine support needs and may be a key component of the dementia literacy assessment toolbox.

## Background

The World Health Organisation defines health literacy as “the cognitive and social skills which determine the motivation and ability of individuals to gain access to, understand and use information in ways which promote and maintain good health” ([1], p10). Evolving beyond the original focus on the fundamental skills of reading, writing and numeracy, health literacy is now understood to include the skills to critically analyse and use information, as well as the skills to engage effectively with the healthcare system and personal health management [2, 3]. It includes having the capacity to obtain and use health services and information, interact with healthcare professionals, care for one’s own health or the health of others, and participate in health-related decision making [4–6]. Low health literacy has been found to contribute to increased susceptibility to poor health outcomes [7–9], exacerbated by limited capacity to use health resources and preventative measures, higher rates of emergency visits, hospitalization, mortality, and linked to poorer understanding of, and lower adherence to, treatment [10].

Several health literacy screening and assessment tools have been developed to measure this evolving social construct of health literacy. Some of these are objective, performance-based assessments of functional skills such as word recognition, comprehension and basic numeracy, while others report an individual’s perceived ability and confidence to undertake health-related tasks, their social resources, and skills [11–15]. While generic health literacy tools are informative, disease-specific literacy tools may be more appropriate in particular contexts [16]. The skills assessed in a disease-specific tool will arguably more closely reflect the actual skills needed in relation to that disease than will a general health literacy tool.

Disease-specific literacy tools may be particularly apt for chronic illnesses that involve complex decisions about care, engaging patients in community-based or self-care contexts [17, 18], and/or where care is likely to be provided by a family member [19, 20]. Dementia is one such condition, as the person living with dementia, in addition to their family members and/or friends, is likely to be involved in both accessing and using health information and care, and making health care decisions on an on-going basis [21–24].

A number of approaches have been taken to assess dementia health literacy; however, like other disease-specific tools, many of these studies focus on measuring knowledge and beliefs about dementia [25–33] without addressing the capacity to access, appraise and apply information. No validated tool is currently available to comprehensively assess the skills required by consumers to navigate and use dementia services, either for themselves or for the people for whom they care.

The current study was informed by responses to discussion boards about support services and resource needs from participants in the Understanding Dementia Massive Open Online Course (UDMOOC) [34], a course designed to meet the need for education about dementia, its causes and care. Through analysing these responses, key issues related to dementia-specific services and health care provision were identified, which in turn informed the development and preliminary validation of the Consumer Access, Appraisal and Application of Services and Information for Dementia (CAAASI-Dem) tool.

We have previously described the Dementia Knowledge Assessment Survey (DKAS), a validated tool to measure dementia knowledge of diverse groups [35, 36]. Here we present the development of the CAAASI-Dem, a complementary tool to measure access, application and appraisal of services and information for dementia, which together with the DKAS is anticipated to enable comprehensive measurement of dementia literacy.

## Methods

The study received ethical approval from the Tasmania Social Sciences Human Research Ethics Committee (Ethics Ref No: H0017429). Potential participants were people who had voluntarily enrolled in the UDMOOC. They were provided with full details of the study via an online link. If they chose to proceed, written informed consent was obtained by the participant selecting “Yes” or “No” to the following online question: I have read the participant information sheet and I consent to participate in this study. Only participants selecting “Yes” were presented with the survey. Participation was entirely voluntary, and participants could withdraw at any time. Responses were anonymised to ensure confidentiality of all study participants. The study was undertaken in three phases: (1) construct identification and item development; (2) scale development and refinement; and (3) preliminary validation.

### Phase 1: construct identification & item generation

In Phase 1, the conceptual boundaries and potential domains of health literacy were identified, followed by the generation of an initial set of items (CAAASI-Dem V1). To capture the full breath of this social construct in the context of dementia, both deductive and inductive methods were employed, including assessment of existing generic health literacy and disease-specific health literacy tools for suitability to be adapted to a dementia context. To identify key dementia service issues important to consumers which might warrant inclusion in the CAAASI-Dem, discussion posts related to services arising from participants in prior iterations of the UDMOOC were collated and common discussion topics were revealed through probabilistic topic modelling analysis using an approach we have applied previously [37]. Structural topic modelling was used to uncover the main themes/topics that underpinned 62,526 UDMOOC participant discussion posts in four UDMOOC offerings between 2014 and 2017. Primary topics were identified by computerised searching for the co-occurrence and exclusivity of words under the assumption that topics can be inferred where distinct collections of words frequently co-occur. Eighty topics were discriminated which captured key issues of importance to consumers in relation to dementia. These topics and the exemplar posts of which they were comprised were subject to interpretive, contextual, qualitative analysis of representative posts to clarify meaning and determine relevance to consumer access, appraisal, and application of dementia-related services and information. Survey items were then drafted which addressed these themes and included in the CAAASI-Dem V1.

The content validity of these initial items in the CAAASI-Dem V1 was then assessed by three experts in the field with extensive experience and expertise in both scale development and dementia subject matter. The experts were invited to review the items for content relevance, comprehensiveness, comprehensibility, and technical quality. Concurrently, respondent debriefings were conducted with six people representative of consumers of dementia services and information, including members of the public who have provided care to family members and health care providers who have provided formal care to people living with dementia. The main purpose of these individual respondent debriefings was to ensure that the intended meaning of each item was fully understood by the target users of the tool. The respondents were asked to initially complete the CAAASI-Dem V1 and subsequently discuss their experience responding to the survey, covering topics such as clarity of instruction and question wording, relevance of response choices, survey format/layout, or reasons for not answering a question. Each of the respondent debriefings lasted between 30 and 45 min. Modifications were subsequently performed in response to the feedback of both the experts and the debriefing respondents, resulting in the second version of the CAAASI-Dem (CAAASI-Dem V2). This was then piloted in two independent samples (Phase 2 and Phase 3).

### Phase 2: scale development and refinement

Phase 2 aimed to further develop and refine the CAAASI-Dem V2. In Phase 2, the CAAASI-Dem V2 was administered to participants of the Understanding Dementia Massive Open Online Course (UDMOOC) at the Wicking Dementia Research and Education Centre, University of Tasmania, in July 2018. Descriptive statistics (frequency, mean, standard deviation, percentage, skewness and kurtosis), other item analysis techniques (item-total and inter-item correlations and Cronbach’s Alpha-if-item-deleted), and qualitative comments were subject to topic modelling analysis as described above to identify the most common topics of concern surrounding item content and construction. Correlation coefficients exceeding 0.5 and 0.3 were regarded as acceptable for item-total and inter-item correlations respectively [38]. Refinement at this stage resulted in a third version of the tool (CAAASI-Dem V3).

### Phase 3: preliminary validation

Phase 3 was conducted with a second cohort of participants in the UDMOOC in February 2019 for factor analysis and preliminary validation of the CAAASI-Dem V3. Factor analysis is primarily used to define the underlying structure of a group of items, hence providing preliminary evidence of construct validity [39]. To assess the suitability of the data for factor analysis, we used Kaiser-Meyer-Olkin (KMO) as a measure of sampling adequacy (>.7) and Barlett’s test of sphericity as a measure of worthwhile correlations among the items within a correlation matrix (*p* < .05) [38]. The correlation matrix was also examined for possible multicollinearity. All items with high correlations (>.80) were assessed, with reference to variance inflation factor, determinant and tolerance indicators. Due to the small proportion of missing data, listwise deletion for missing data was employed.

For factor extraction, an initial exploratory factor analysis using Principal Axis Factoring (PAF) was performed, as this method entails no distributional assumptions [40]. The PAF unrotated factor matrix provided a preliminary estimate of the factor numbers and indicated the potential for data reduction.

Factor retention was then determined using a combination of criteria, including Kaiser–Guttman rule (i.e. Eigenvalues greater than 1) [41], the scree test (i.e. change of slope) [42], and parallel analysis [43, 44]. Interpretability of the factors was also taken into consideration.

After the factor number was identified, we conducted another PAF analysis with Oblimin (oblique) rotation, which allows factors to be correlated and which is considered most relevant for exploring theoretically meaningful factors [38]. Considering the large sample, items were retained if they had significant factor loadings (.3 or higher) on one factor and no/low loadings on all other factors (a difference of at least .2 between the loadings). Items with cross-loading and low communalities were considered for deletion. To be retained in the final model/solution, a factor needed to have at least three items with significant factor loadings [45]. The extracted factors were then descriptively labelled.

The internal consistency of each of the factors in the final model was evaluated using Cronbach’s alpha coefficients. A coefficient of 0.7 or higher was indicative of an internally consistent survey [46, 47].

All quantitative data analyses were conducted using SPSS version 22 (IBM Corp., Armonk, NY) and the R statistical computing environment.

## Results

### Phase 1: construct identification & item generation

Health literacy tools which comprehensively address multiple aspects of health literacy and provide foundation questions which could be adapted to a dementia context were identified. From available tools, the Health Literacy Questionnaire (HLQ) [13], the All Aspects of Health Literacy Scale (AAHLS) [12], and the European Health Literacy Survey Questionnaire (HLS-EU-Q47) [14] were selected to inform development of the first version of the CAAASI-Dem to cover concepts of: a) access to dementia health care support; b) adequacy, quality and relevance of information; c) social support; and d) engagement with the health care system. Analysis of consumer posts in the UDMOOC identified the following key issues for consideration in the first version of the tool: use of the major support and referral service (e.g. in Australia: Dementia Australia); use of the primary consumer interface with health services (e.g. in Australia: MyAged Care); modes of access to information including internet, phone and face to face; and key areas of dementia information need including nutrition, exercise, depression, anxiety, mobility, continence, behaviour, social engagement, sleep, community support, transitional needs, and isolation.

The first draft of the CAAASI-Dem (CAAASI-Dem V1) had a pool of 70 items, including 22 items adapted from a generic health context to a dementia context from the HLQ [13], 4 adapted from the AAHLS [12], and 44 items generated through content analysis of UDMOOC posts. Questions from HLS-EU-Q47 were not selected for adaptation to the dementia context as the domains were addressed in other items and it is a tool with a more generic application [14]. The included items address a range of cognitive, communicative, and social skills required in performing different tasks of finding, understanding, appraising, and using dementia health information and services. As a self-report tool, the CAAASI-Dem V1 measures people’s perceived ability in performing those health-related tasks on either a 5-point Likert scale (Not at all confident – Extremely confident; or Strongly agree – Strongly disagree) or a binary scale (Yes – No), with an additional section containing demographic questions.

As a result of the content validity assessment by both experts and debriefing respondents, five items were dropped from the initial pool of items because they were regarded as irrelevant to the social construct of health literacy as it applies to dementia. Instructions and eight items were reworded to improve comprehensibility and address the feedback about ambiguity, inconsistency, wordiness, and lack of clarity. Regarding the response options, ‘Neutral’ was replaced by ‘Neither agree or disagree’, and ‘Not applicable’ was replaced by ‘Not relevant to me now’. The binary response option of four items was also changed to a 5-point Likert scale (Strongly agree – Strongly disagree). The resultant CAAASI-Dem V2 included 65 items. For Phase 2 testing, two open-ended questions and a Yes-No question were added to seek participant feedback that might further inform the survey development.

### Phase 2: scale development and refinement

A total of 1412 participant responses were analysed in Phase 2. They comprised a predominantly female population who had voluntarily enrolled in the UDMOOC, consented to participate in the CAAASI-Dem study and answered *yes* to the question: Have you ever looked for dementia-related services for yourself or someone else? Within this group were a high frequency of participants who had provided care for a family member or friend with dementia (92.2%). The full demographic profile is shown in Table 1.

**Table 1:** Demographic profile of participants in Phase 2 and Phase 3

	Phase 2	Phase 3
	(*n* = 1412)	(*n* = 3146)
Age		
Mean (SD)	51.2 (13.1)	46.1 (14.2)
Not indicated	49 (3.5%)	178 (5.7%)
**Sex**		
Female	1232 (87.3%)	2709 (86.1%)
Male	162 (11.5%)	397 (12.6%)
Not indicated	18 (1.3%)	40 (1.3%)
**English as a first language**		
No	213 (15.1%)	515 (16.4%)
Yes	1186 (84.0%)	2594 (82.5%)
Not indicated	13 (0.9%)	37 (1.2%)
**Highest level of completed education**		
Primary school	1 (0.1%)	5 (0.2%)
Secondary school (years 11–12)	121 (8.6%)	247 (7.9%)
Secondary school (years 7–10)	85 (6.0%)	149 (4.7%)
Certificate or apprenticeship (including Cert 2, 3 or 4)	221 (15.7%)	584 (18.6%)
Diploma / Associate degree	290 (20.5%)	649 (20.6%)
Bachelor’s degree	337 (23.9%)	847 (26.9%)
Higher University degree (Hons, Grad. Dip, Masters or PhD)	280 (19.8%)	539 (17.1%)
Not indicated	77 (5.5%)	126 (4.0%)
**Prior dementia education**		
No	612 (43.3%)	1284 (40.8%)
Yes	770 (54.5%)	1816 (57.7%)
Not indicated	30 (2.1%)	46 (1.5%)
**Do you have dementia?**		
No	1245 (88.2%)	3046 (96.8%)
Prefer not to say	4 (0.3%)	2 (0.1%)
Unsure	138 (9.8%)	52 (1.7%)
Yes	18 (1.3%)	5 (0.2%)
Not indicated	7 (0.5%)	41 (1.3%)
**Family member with dementia**		
No	275 (19.5%)	1461 (46.4%)
Yes	1131 (80.1%)	1650 (52.4%)
Not indicated	6 (0.4%)	35 (1.1%)
**Other close associate with dementia**		
No	424 (30.0%)	1608 (51.1%)
Yes	974 (69.0%)	1490 (47.4%)
Not indicated	14 (1.0%)	48 (1.5%)
**Provided unpaid care for a person with dementia**		
No	110 (7.8%)	2261 (71.9%)
Yes	1302 (92.2%)	858 (27.3%)
Not Indicated	0 (0%)	27 (0.9%)
**Experience as a paid carer**		
No	576 (40.8%)	877 (27.9%)
Yes	820 (58.1%)	2255 (71.7%)
Not Indicated	16 (1.1%)	14 (0.4%)
**Australian resident**		
No	703 (49.8%)	1509 (48.0%)
Yes	709 (50.2%)	1637 (52.0%)

A close examination of the descriptive statistics and qualitative feedback led to 34 items being removed from the CAAASI-Dem V2. In these cases, responses were highly dependent on the consumer’s individual experience of, and need for, those specific services (for example: continence, nutrition, or mobility) rather than their capacity to access, apply and appraise them. Eleven items were also revised to improve clarity, in line with the qualitative feedback from participants which indicated that the response perspective was not always clear (for example, respondents may have had multiple and different experiences as family or professional carers, which made their choice of response options less certain). The resulting CAAASI-Dem V3 had a total of 31 items, which was piloted in Phase 3.

### Phase 3: preliminary validation

Phase 3 attracted the voluntary participation of 3146 UDMOOC respondents. Unlike Phase 2, analysis in this phase included all participants irrespective of their prior history of seeking dementia specific services, thus the participant profile was typical of those who enrol in the UDMOOC [48]. The demographic profile is shown in Table 1.

Initial item analysis techniques were again employed to identify other items that needed to be removed or modified and to determine the data distribution to inform the selection of factor analysis methods. The inter-item correlations indicated possible redundancy, with two items (Filling out forms online and Filling out forms on paper) being highly correlated (.93). Therefore, one item (Filling out forms online) was removed from the subsequent exploratory factor analysis. An examination of skewness and kurtosis, and the Shapiro-Wilk W test results (*p* < .001) indicated a departure from univariate normality for all items, hence the rejection of multivariate normality [49]. Therefore, PAF was chosen for factor analysis.

### Exploratory factor analysis

The KMO value was .95 and the Barlett’s test was significant (X^2^ = 84,210.0; *p* < .001), which indicated the suitability of the data for factor analysis. There was no indication of multicollinearity, and no items had problematically low correlations (*r* < .30) with many other items.

The initial PAF yielded six factors with eigenvalues greater than 1.0, which collectively explained 74.7% of the total variance. The eigenvalue of the sixth factor (1.04) was very close to the 1.0 cut-off criterion. The scree plot and parallel analysis results indicated five factors should be extracted. Therefore, the second PAF with Oblimin rotation was conducted with a fixed number of five factors.

One item exhibited cross-loading (Arranging health care services) and three items (Finding information about dementia on the internet, face-to-face, or by phone) had low communalities (< .5). These items were considered for sequential elimination, taking into account their theoretical contribution to the construct of dementia literacy. The PAF with a new factor solution was rerun each time an item was eliminated, and four items were eventually dropped from the model. The final factor structure and loadings of items to each factor are shown in Table 2.

**Table 2:** Factor structure and item loadings

	Factor					Communality
Item content	1	2	3	4	5	
Deciding if dementia health information is relevant to me	.917					.754
Knowing whether to believe information about dementia	.864					.712
Understanding the health care advice that I am given about dementia	.851					.769
Questioning advice on dementia given to me by a healthcare provider	.810					.717
Comparing dementia health information from different sources	.800					.710
Discussing dementia with a healthcare provider by myself	.761					.743
Reading information from a healthcare provider by myself	.684					.686
Discussing very sensitive and personal issues about dementia with healthcare providers	.681					.687
Finding dementia health information by myself	.636					.597
I have enough information about dementia to plan for future needs		.880				.617
I have enough information about dementia to help me deal with current needs		.803				.744
I have good quality information about dementia		.676				.625
I can rely on at least one healthcare provider to help when there is urgent need		.593				.529
I have a least one healthcare provider who can give me advice about dementia-related healthcare needs		.582				.533
Knowing which healthcare services are available		.440				.613
Working out what dementia services may be needed in the future		.391				.615
Finding dementia related health services		.386				.629
If I need help, I have people I can call			.941			.885
There are people I can spend time with			.870			.800
I have support from my community			.668			.547
Accessing respite care				.933		.861
Organising an aged care assessment				.906		.750
Organising an advance care plan				.847		.750
Getting myself to healthcare appointments					−.888	.831
Getting others to healthcare appointments					−.820	.761
Filling out forms on paper					−.608	.613

Extraction method: Principal Axis Factoring, Oblimin rotation with Kaiser Normalization. Factor loadings less than .30 are not shown, and variables are sorted by loadings on each factor.

As demonstrated, the final CAAASI-Dem was found to be multidimensional, with five factors labelled: *Evaluation and engagement; Readiness; Social supports; Specific dementia services*; and *Practical aspects*. The five-factor solution explained 69.7% of the total variance. A summary of the factors is shown in Table 3.

**Table 3:** Factor summary

Factor	Factor label	Number of items	Cronbach’s Alpha	Definition
Factor 1	Evaluation & engagement	9	.953	Confidence in critically and independently engaging with dementia-related information and advice from a range of sources
Factor 2	Readiness	8	.911	Confidence in both knowledge of and ability to access a range of appropriate healthcare information and supports over the course of the dementia trajectory
Factor 3	Social supports	3	.887	Personal assessment of and access to available human and community supports
Factor 4	Specific dementia services	3	.926	Confidence in organizing and accessing specific dementia-related services
Factor 5	Practical aspects	3	.888	Confidence in physically navigating elements of the health care system

### Internal consistency reliability

As shown in Table 3, the Cronbach’s alpha scores of each of the factors were: .953 for *Evaluation and engagement*, .91 for *Readiness*, .89 for *Social supports*, .93926 for *Specific dementia services*, and .89 for *Practical aspects*. These scores all well exceeded the acceptability criterion of .7. The subscale inter-item correlations were all greater than .30, and all item-subscale correlations exceeded .50. Examination of the Cronbach’s Alpha-if-item-deleted suggested that deleting any of the items would not result in a markedly higher (>.04) alpha value. The results indicated a high internal consistency or inter-correlation between the items within each factor.

## Discussion

Using a multi-stage process, incorporating both quantitative and qualitative methods, we have developed the first tool to measure key aspects of consumer health literacy specific to dementia contexts. The final 26-item CAAASI-Dem comprises five factors: *Evaluation and engagement*, *Readiness, Social supports, Specific dementia services*, and *Practical aspects*. Factor analytic and other statistical findings suggest that the multi-dimensional CAAASI-Dem has adequate internal consistency reliability and construct validity for this population of participants in the UD-MOOC, who comprise a cross section of individuals with an interest in dementia. While carers comprise a large proportion of our respondents, consumers is a broader term used to collectively describe anyone seeking, or needing, information and services about dementia and includes people living with dementia, their families, informal carers, paid carers, and health care providers. Our participant cohort includes these groups; thus, the term consumer was used to collectively describe this population.

The five-factor structure of the CAAASI-Dem is different to existing questionnaires for generic or disease-specific health literacy assessment, reflecting the unique aspects, elements and challenges dementia presents. The three levels of health literacy defined by Nutbeam and a feature of the AAHLS [12], being functional, communicative and critical health literacy – are not discrete factors of this dementia-specific tool. Firstly, the cognitive and communicative skills needed to obtain, understand, and appraise health information were related to one single underlying dimension or factor, which we have labelled *Evaluation and engagement*. This may reflect the inherent inter-relatedness of these skills and the context in which they are employed by a person to process or make sense of health information, often for someone for whom they care, and either independently or interactively with health professionals [50]. Secondly, and not unexpectedly, the skills needed for engaging with specific services, namely organizing an aged care assessment, an advance care plan, or respite care, which are often undertaken on behalf of a third party, constituted an empirically distinct dimension in our instrument (*Specific dementia services*) and comprise key issues raised by UDMOOC participants. This factor distinctly differentiates the newly developed CAAASI-Dem from other general health literacy measures, in that it acknowledges the specific role of the carer. This aligns with the Health Literacy of Caregivers Scale-Cancer (HLSC-C) which identifies a separate domain related to understanding the healthcare system and determining the need for services on behalf of the person with the disease [20]. *Readiness* is a unique emerging dimension of relevance to dementia. This factor highlights the importance of being ready or well-prepared for the dementia trajectory itself and the dynamic nature of support needs this entails, for both the person living with dementia, their family, the healthcare system, and the wider community [51]. Specifically, *Readiness* describes a group of skills needed to assess and respond to current needs and to anticipate future needs related to dementia information and services; this includes the ability to navigate systems, evaluate the information or services readily available to them, and make appropriate plans, thus comprising critical and functional components.

Consistent with the 9-factor scale by Osborne et al. [13], the 8-factor scale by Jordan et al. [11], and the HLCS-C [20], social support is an empirically distinct dimension of the CAAASI-Dem. However, unlike the generic health literacy tools which focus on social support for health management and access, in the context of dementia, social support from community and friends has deeper implications, especially for carer resilience [52]. Social connectedness of the person living with dementia and their carer can be significantly affected by dementia and can lead to loneliness and isolation [53], which may impact not only on health status but the capacity of both the person living with dementia and their carer to engage with the healthcare system [54].

As a whole, the five factors and their 26 constituent items in the CAAASI-Dem align with the functional, critical and communicative aspects of dementia health literacy, which when complemented with existing measures of dementia knowledge such as DKAS, will represent a comprehensive approach to measuring dementia-specific health literacy. This newly developed measure could be utilised as a screening tool to collect baseline population-wide information about consumer engagement with dementia services, and to identify possible gaps and needs in relation to such services, which would enable the tailoring of dementia health information and services to specific groups of consumers. In addition, use of the CAAASI-Dem could help inform policies and educational interventions, as well as measure the impact of dementia-related health promotion initiatives.

### Limitations

The analysis was conducted using data from samples recruited from a single institution from participants in a free educational massive open online course. Consequently, respondents were restricted to those with internet access and with a personal interest in dementia. A high proportion of participants in each phase of this study identified as carers, hence the CAAASI- Dem might be described as assessing these aspects of dementia literacy from a carer perspective, however consumers of dementia information extend beyond those providing direct care in either a formal or informal sense. While the demographic profile of participants was representative of those needing or seeking dementia-related services, and the future application of the tool is both intended for such a cohort and likely to be delivered on-line, additional testing of the tool should be undertaken in a non-UDMOOC participant cohort. This may illuminate the access, appraisal and application issues of a less digitally literate population and allow exploration of differing needs in relation to stage of disease and care relationships. Because many health-related services are currently delivered online, those with poor digital literacy may have different and under-served support requirements. Exploring non UDMOOC participants may expose comparative differences in items such as filling out forms online or on paper which has implications for ongoing delivery of information and support.

As a self-reported measure, the CAAASI-Dem has the inherent limitation of potential bias due to social desirability (although such bias has been minimised by the survey being anonymous and voluntary).

The items generated from participant posts in the UDMOOC referring to support and services for dementia gave rise to a large number of individual topics related to often specific support needs. Because of the predominantly Australian profile of respondents, many of these were particular to the Australian context. For future application to an international cohort, more generic terms referring to support services, needs assessment and respite may need to be considered.

During the process of item reduction, many specific items such as those related to support needs for issues such as dysphagia, incontinence, and behaviours were eliminated. It is recognised that the need for such specific services may become more important to people living with dementia and their carers as the condition progresses. While these items are not included in the final CAAASI-Dem, they should be addressed as part of a companion checklist to gain insight into specific service and information gaps. Consideration must also be given to the high frequency of comorbidities that present with dementia. While the purpose of this tool was to focus on dementia specific information and services, further work should examine the changing information and service needs for other health conditions over the dementia trajectory.

The dementia-relevant services which were retained in the tool comprise advance care planning, respite services and aged care assessments. The primary access point for information on the aged care system, needs assessment and access to services in Australia is an online website (https://www.myagedcare.gov.au). Inability to access or navigate this website would impede engagement with key support services such as respite care. Given the CAAASI-Dem was also designed for online use, face to face or paper versions may be required to illuminate the needs of those not able to confidently use online technology.

This study produced favourable psychometric properties, however further research is needed to further refine and validate the CAAASI-Dem. Different aspects/types of reliability (e.g. test-retest) and validity (e.g. content, criterion, and construct) will be examined.

## Conclusion

In this paper we have reported on the development of a dementia-specific tool designed to comprehensively assess the skills required by consumers to navigate and use dementia services and information, either for themselves or for the people for whom they care. This instrument, the Consumer Access, Appraisal and Application of Services and Information for Dementia (CAAASI-Dem), shows good psychometric properties, notwithstanding limitations of sample and the need for further testing. The tool is designed to be used in conjunction with the Dementia Knowledge Assessment Scale (DKAS) to enable a comprehensive model of measuring dementia literacy. The complexities of the dementia syndrome and the projected associated needs for support and care are such as to render this a dementia health literacy indicator of particular utility into the future.

## Data Availability

The datasets used and/or analysed during the current study are available from the corresponding author on reasonable request.

## Abbreviations

CAAASI-Dem: Consumer Access, Appraisal and Application of Services and Information for Dementia
DKAS: Dementia Knowledge Assessment Survey
HLSC-C: Health Literacy of Caregivers Scale-Cancer
HLS-EU-Q47: European Health Literacy Survey Questionnaire
HLQ: Health Literacy Questionnaire
KMO: Kaiser-Meyer-Olkin
PAF: Principal Axis Factoring
UDMOOC: Understanding Dementia Massive Open Online Course

## References

[1] World Health Organisation (1998). Health promotion glossary.

[2] Chinn D (2011). Critical health literacy: a review and critical analysis. Soc Sci Med.

[3] Nutbeam D, McGill B, Premkumar P (2018). Improving health literacy in community populations: a review of progress. Health Promot Int.

[4] Nutbeam D (2000). Health literacy as a public health goal: a challenge for contemporary health education and communication strategies into the 21st century. Health Promot Int.

[5] Nutbeam D (2008). The evolving concept of health literacy. Soc Sci Med.

[6] Batterham RW, Hawkins M, Collins PA, Buchbinder R, Osborne RH (2016). Health literacy: applying current concepts to improve health services and reduce health inequalities. Public Health.

[7] Kickbusch IS (2001). Health literacy: addressing the health and education divide. Health Promot Int.

[8] Carmona RH (2006). Health literacy: a national priority. J Gen Intern Med.

[9] Zarcadoolas C, Pleasant A, Greer D (2006). Advancing health literacy: a framework for understanding and action.

[10] Berkman ND, Sheridan SL, Donahue KE, Halpern DJ, Crotty K (2011). Low health literacy and health outcomes: an updated systematic review. Ann Intern Med.

[11] Jordan JE, Buchbinder R, Briggs AM, Elsworth GR, Busija L, Batterham R, Osborne RH (2013). The health literacy management scale (HeLMS): a measure of an individual's capacity to seek, understand and use health information within the healthcare setting. Patient Educ Couns.

[12] Chinn D, McCarthy C (2013). All aspects of health literacy scale (AAHLS): developing a tool to measure functional, communicative and critical health literacy in primary healthcare settings. Patient Educ Couns.

[13] Osborne RH, Batterham RW, Elsworth GR, Hawkins M, Buchbinder R (2013). The grounded psychometric development and initial validation of the health literacy questionnaire (HLQ). BMC Public Health.

[14] Sorensen K, Van den Broucke S, Pelikan JM, Fullam J, Doyle G, Slonska Z, Kondilis B, Stoffels V, Osborne RH, Brand H (2013). Measuring health literacy in populations: illuminating the design and development process of the European health literacy survey questionnaire (HLS-EU-Q). BMC Public Health.

[15] Kiechle ES, Bailey SC, Hedlund LA, Viera AJ, Sheridan SL (2015). Different measures, different outcomes? A systematic review of performance-based versus self-reported measures of health literacy and numeracy. J Gen Intern Med.

[16] Tique JA, Howard LM, Gaveta S, Sidat M, Rothman RL, Vermund SH, Ciampa PJ (2017). Measuring health literacy among adults with HIV infection in Mozambique: development and validation of the HIV literacy test. AIDS Behav.

[17] Dumenci L, Matsuyama R, Riddle DL, Cartwright LA, Perera RA, Chung H, Siminoff LA (2014). Measurement of cancer health literacy and identification of patients with limited cancer health literacy. J Health Commun.

[18] Yeh J-Z (2018). Wei C-j, Weng S-f, Tsai C-y, Shih J-h, Shih C-l, Chiu C-h: **disease-specific health literacy, disease knowledge, and adherence behavior among patients with type 2 diabetes in Taiwan**. BMC Public Health.

[19] Yuen EY, Knight T, Dodson S, Ricciardelli L, Burney S, Livingston PM (2014). Development of the health literacy of caregivers scale - Cancer (HLCS-C): item generation and content validity testing. BMC Fam Pract.

[20] Yuen E, Knight T, Dodson S, Chirgwin J, Busija L, Ricciardelli LA, Burney S, Parente P, Livingston PM (2018). Measuring cancer caregiver health literacy: validation of the health literacy of caregivers scale–Cancer (HLCS-C) in an Australian population. Health & social care in the community.

[21] Groen-van de Ven L, Smits C, Span M, Jukema J, Coppoolse K, de Lange J, Eefsting J, Vernooij-Dassen M (2018). The challenges of shared decision making in dementia care networks. Int Psychogeriatr.

[22] Miller LM, Whitlatch CJ, Lyons KS (2014). Shared decision-making in dementia: a review of patient and family carer involvement. Dementia.

[23] Harland J, Bath PA, Wainwright A, Seymour J (2017). Making sense of dementia: a phenomenographic study of the information needs and behaviours of people with dementia at diagnosis. Aslib J Inf Manag.

[24] Peterson K, Hahn H, Lee AJ, Madison CA, Atri A (2016). In the information age, do dementia caregivers get the information they need? Semi-structured interviews to determine informal caregivers' education needs, barriers, and preferences. BMC Geriatr.

[25] Low LF, Anstey KJ (2009). Dementia literacy: recognition and beliefs on dementia of the Australian public. Alzheimers Dement.

[26] Sun F, Gao X, Coon DW (2013). Perceived threat of Alzheimer’s disease among Chinese American older adults: the role of Alzheimer’s disease literacy. J Gerontol Series B: Psychological Sciences & Social Sciences.

[27] Noble JM, Hedmann MG, Williams O (2015). Improving dementia health literacy using the FLOW mnemonic: pilot findings from the old SCHOOL hip-hop program. Health Educ Behav.

[28] Diamond AG, Woo BK (2014). Duration of residence and dementia literacy among Chinese Americans. Int J Soc Psychiatry.

[29] Loi SM, Lautenschlager NT (2015). Dementia literacy in older adults. Asia Pac Psychiatry.

[30] Low LF, Anstey KJ, Lackersteen SM, Camit M, Harrison F, Draper B, Brodaty H (2010). Recognition, attitudes and causal beliefs regarding dementia in Italian, Greek and Chinese Australians. Dement Geriatr Cogn Disord.

[31] Millard FB, Kennedy RL, Baune BT (2011). Dementia: opportunities for risk reduction and early detection in general practice. Aust J Primary Health.

[32] Zhang H, Loi SM, Zhou S, Zhao M, Lv X, Wang J, Wang X, Lautenschlager N, Yu X, Wang H (2017). Dementia literacy among community-dwelling older adults in urban China: a cross-sectional study. Front Public Health.

[33] Valle R, Yamada A-M, Matiella AC (2006). Fotonovelas: a health literacy tool for educating Latino older adults about dementia. Clin Gerontol.

[34] King C, Doherty K, Kelder J-A, McInerney F, Walls J, Robinson A (2014). Vickers J: **‘fit for purpose’: a cohort-centric approach to MOOC design**. Int J Educ Technol High Educ.

[35] Annear MJ, Toye C, Eccleston C, McInerney F, Elliott KE, Tranter BK, Hartley T, Robinson A (2015). Dementia knowledge assessment scale: development and preliminary psychometric properties. J Am Geriatr Soc.

[36] Annear MJ, Toye C, Elliott KJ, McInerney F, Eccleston C, Robinson A (2017). Dementia knowledge assessment scale (DKAS): confirmatory factor analysis and comparative subscale scores among an international cohort. BMC Geriatr.

[37] McInerney F, Doherty K, Bindoff A, Robinson A, Vickers J (2018). How is palliative care understood in the context of dementia? Results from a massive open online course. Palliat Med.

[38] Hair JF, Black WC, Babin BJ, Anderson RE (2014). Multivariate data analysis 7edn.

[39] Hahs-Vaughn DL (2017). Applied multivariate statistical concepts.

[40] Fabrigar LR, Wegener DT, MacCallum RC, Strahan EJ (1999). Evaluating the use of exploratory factor analysis in psychological research. Psychol Methods.

[41] Kaiser HF (1960). The application of electronic computers to factor analysis. Educ Psychol Meas.

[42] Cattell RB (1966). The scree test for the number of factors. Multivar Behav Res.

[43] Hayton JC, Allen DG, Scarpello V (2004). Factor retention decisions in exploratory factor analysis: a tutorial on parallel analysis. Organ Res Methods.

[44] Horn JL (1965). A rationale and test for the number of factors in factor analysis. Psychometrika.

[45] Costello AB, Osborne JW (2005). Best practices in exploratory factor analysis: four recommendations for getting the most from your analysis. Pract Assess Res Eval.

[46] Nunnally J, Bernstein L (1994). Psychometric theory.

[47] Tavakol M, Dennick R (2011). Making sense of Cronbach’s alpha. Int J Med Education.

[48] Eccleston C, Doherty K, Bindoff A, Robinson A, Vickers J, McInerney F (2019). Building dementia knowledge globally through the Understanding Dementia Massive Open Online Course (MOOC). npj Science of Learning.

[49] Looney SW (1995). How to use tests for univariate normality to assess multivariate normality. Am Stat.

[50] McCormack L, Thomas V, Lewis MA, Rudd R (2017). Improving low health literacy and patient engagement: a social ecological approach. Patient Educ Couns.

[51] Hodgson N, Black B, Johnston D, Lyketsos C, Samus Q (2014). Comparison of unmet care needs across the dementia trajectory: findings from the maximizing independence at home study. J Geriatr Palliat Care.

[52] Jones SM, Woodward M, Mioshi E. Social support and high resilient coping in carers of people with dementia. Geriatr Nurs. 2019. 10.1016/j.gerinurse.2019.05.011 31178232

[53] Moyle W, Kellett U, Ballantyne A, Gracia N (2011). Dementia and loneliness: an Australian perspective. J Clin Nurs.

[54] Vernooij-Dassen M, Moniz-Cook E, Jeon YH (2018). Social health in dementia care: harnessing an applied research agenda. Int Psychogeriatr.

